# Enteric parasitic infection disturbs bacterial structure in Mexican children with autoantibodies for type 1 diabetes and/or celiac disease

**DOI:** 10.1186/s13099-020-00376-3

**Published:** 2020-08-11

**Authors:** Ana M. Calderón de la Barca, Reyna S. Castillo-Fimbres, María Esther Mejía-León, Luis Quihui-Cota, Adrián Ochoa-Leyva, Sandra V. Aguayo-Patrón

**Affiliations:** 1 Dept. Nutrición, Centro de Investigación en Alimentación y Desarrollo, A.C., Astiazarán Rojas No. 46, Hermosillo, 83304 Sonora Mexico; 2 Facultad de Medicina Mexicali, Universidad Autónoma de Baja California, Dr. Humberto Torres Sanginés S/N Centro Cívico, Mexicali, 21000 B.C. Mexico; 3 Dept. Microbiología Molecular, Instituto de Biotecnología, Universidad Nacional Autónoma de México, Cuernavaca. Av. Universidad 2001, Col. Chamilpa, Cuernavaca, 62210 Morelos Mexico; 4 Universidad del Valle de México, Hermosillo, Son. Mexico

**Keywords:** Enteric pathogens, *Cryptosporidium*, *Cyclospora*, *Akkermansia*, Celiac disease, Type 1 diabetes

## Abstract

**Background:**

Intestinal bacterial dysbiosis and increased gut permeability are associated with higher risk of developing type 1 diabetes (T1D) or celiac disease (CD). There is a lack of information on parasitism involved in gut disturbance of predisposed children. We evaluated the effect of enteropathogenic parasites (*Cryptosporidium* spp., *Cyclospora* spp. *G. lamblia*, and *Blastocystis* spp.) on the bacterial structure of feces from children with autoantibodies for T1D or CD. Participants included 37 children under 18 years of age, from whom stools were analyzed for enteric parasites by qPCR and 22/37 for bacterial profile by sequencing the V3–V4 region of the 16s rRNA gene. Dietary, clinical, and socioeconomic data was recorded.

**Results:**

Pathogens parasitized 28/37 participants, *Cryptosporidium* spp. was the most prevalent (62.2%), followed by both *Cyclospora cayetanensis* and *Blastocystis* spp (37.8%). There were no dietary differences (*p *> 0.05) attributable to parasitism. Co-infected participants with *Cryptosporidium* and *Cyclospora did not* differ (p = 0.064) from non-infected participants in bacterial alpha phylogenetic diversity. The same parasites’ co-infection was associated with a decreased abundance of the Ruminococaceae (p = 0.04) and Verrucomicrobioceae families, of the *Akkermansia* genus (p = 0.009). There was a lower Firmicutes/Bacteroidetes ratio (p = 0.02) in infected than in uninfected participants.

**Conclusions:**

*Cryptosporidium* and *Cyclospora* affected the bacterial structure at family and genus levels, decreasing the ratio between Firmicutes and Bacteroidetes in children with auto-antibodies for T1D or CD, which could increase the risk of illness onset.

## Background

Twenty-five years ago, studies about the pathogenesis of celiac disease (CD), an autoimmune enteropathy triggered by wheat consumption, proposed that gut health disturbance influences its onset [30]. Nowadays, the disruption of tight junctions by bacterial-derived products and/or gliadin peptides, which affects the intestinal permeability, is related to the development of CD [11, 23]. Gut health has also been associated to the onset of type 1 diabetes (T1D), through the identification of three gut dynamic components involved in its pathogenesis: the mucosal immunity, the microbiota and the individual’s diet [33, 39]. T1D is another autoimmune disease similar to CD in predisposition factors, characterized by the destruction of the insulin-producing β-cells in pancreas. It has been related to a deficiency of short-chain fatty acids-producing bacteria in the gut, as well as to an increased intestinal permeability [20, 36].

CD and T1D are the two most frequent autoimmune diseases in childhood. The International Diabetes Federation estimates that, globally, more than one million children and adolescents under the age of 19 have T1D, with an annual incidence of almost one hundred thirty thousand new cases [25]. Both T1D and CD incidence have increased in recent decades, mainly attributed to common environmental or pathophysiological mechanisms. Thus, presenting T1D increases the risk of developing CD, in a range between 2.5 and 16.4% in different populations, several times its global prevalence of 2–3% [13].

The intestinal bacterial imbalance (dysbiosis) and the enteropathogenic parasites that affect the gut barrier, have been independently associated with increased risk of developing T1D or CD in genetically predisposed children [9, 23, 35]. However, there is no information regarding the effect of enteropathogenic parasites’ infections on bacterial dysbiosis or vice versa, in children developing autoimmunity. Therefore, our study had two objectives. The first was to evaluate the prevalence of enteropathogenic parasites in children under 18 years with autoantibodies associated with T1D or CD, pre-symptomatic or already diagnosed. The second one was to look for the effect of the enteropathogenic parasites’ infection in the fecal bacterial structure in children developing autoimmunity associated to T1D and/or CD.

## Results

### Characteristics of participants

Participants were 37 children, 25 of them were girls. Their mean age was 10.93 ± 3.02 years, weight 39.52 ± 17.2 kg and height 141.44 ± 16.05 cm (values are means ± standard deviations). Socioeconomic status was medium for 26 of the participants, with sufficient resources to meet all the family requirements; but 11 belonged to families with poor household circumstances and had access to only some public services.

### Autoantibodies and diagnosis status for T1D and CD

All participants presented antibodies associated with T1D or CD because it was an inclusion criterion. Sixteen of 37 participants had a diagnosis for T1D and 2 for CD; 4 of them had autoimmunity (at least two different autoantibodies) and 15 presented only one type of antibodies related to T1D. Ten of the 18 diagnosed participants were at onset of the illness, while one of them had 12 months of evolution and the remaining 7 were diagnosed with T1D at least for 24 months. All of participants had a high genetic risk for T1D or CD, presenting HLA DQ2, DQ8 haplotypes, or any combination of related alleles in their genotype.

### Detection and identification of enteropathogenic parasites

Twenty-eight of 37 participants were infected by enteropathogenic parasites. *Cryptosporidium* spp. showed the highest prevalence (62.2%), followed by *Cyclospora* spp. and *Blastocystis* spp. (37.8%). Table 1 shows prevalence of all detected intestinal parasites.

**Table 1:** Enteric parasites detection in participants with T1D or CD associated antibodies (n = 37)

Pathogenic Parasites	Positive samples (n = 28)	Prevalence (%)
*Cryptosporidium* spp.	23	62.2
*Cyclospora* spp.	14	37.8
*Blastocystis* spp.	14	37.8
*Giardia lamblia*	7	18.9
*Entamoeba histolytica*	1	2.7

### Dietary evaluation

There were no significant differences (*p* > 0.05) for energy, macronutrient nor dietary fiber consumption between infected and uninfected participants with enteropathogenic parasites. The energy intake (mean ± standard deviation) of the group with enteric pathogen parasites (n = 28) was 1837.6 ± 919 kcal/day, with 33.8% lipids, 14.6% proteins and 53.2% carbohydrates. In the uninfected group (n = 9), energy intake was 1667.7 ± 489 kcal/day, with 37.2% lipids, 15.3% protein and 48.7% carbohydrates. Almost half of the total children (49%) consumed a healthy dietary pattern, while 35% of them had an ultra-processed pattern, and 16% received a poor dietary pattern.

### Fecal bacterial microbiota analysis

Fecal bacterial microbiota sequencing was done in samples of 22 participants, 18 infected with enteropathogenic parasites and 4 uninfected, whose characteristics are shown in Table 2. Infected participants had at least one parasite; 15 with *Cryptosporidium* spp. 9 with *Cyclospora* spp. 6 with *G. lamblia* and 12 with *Blastocystis* spp. Only 3 of the 22 analyzed samples were from 2 children at the onset of CD, and the third one coursed with more than 2 years of T1D evolution. Seven children presented more than one autoantibody’s type, and the remaining 15 children were only positive for insulin autoantibodies (IAA).

**Table 2:** General characteristics of the participants in the fecal microbiota analysis (n = 22)

	Infected with enteropathogenic parasites, (n = 18)	Non-infected with enteropathogenic parasites (n = 4)
Sex (F/M)	12/6	4/0
Age (years ± SD)	9.88 ± 2.10	10 ± 0.81
BMI (kg/m^2^ ± SD)	17.7 ± 3.58	21.36 ± 10.75
Haplotype		
HLA DQ2	9	2
HLA DQ8	3	0
Other risk genotype^a^	6	2
Autoanti-bodies (positives/n)		
Anti-IAA	15/18	3/4
Anti-GAD	1/18	0/4
Anti-IA2	1/18	0/4
Anti-Gd	5/18	1/4
Anti-TG	3/18	1/4
T1D diagnosis (n)	1	0
CD diagnosis (n)	1	1
Diet		
Energy (kcal/d ± SD)	1636.3 ± 444.4	1746.7 ± 598.3
Carbohydrates (% ± SD)	57.0 ± 8.6	61.6 ± 14.6
Proteins (% ± SD)	12.3 ± 3.7	11.2 ± 2.9
Lipids (% ± SD)	32.5 ± 6.5	28.9 ± 14.2

Independent samples t tests were performed, p ≤ 0.05. No significant differences were found between groups

*F* female, *M* male, *SD* standard deviation, *n* sample size, *T1D* type 1 diabetes, *CD* celiac disease

^a^Participants with one or two HLA risk alleles (DQA1 0501, DQB1 0201, DQA1 0301, DQB1 0302)

After using quality filters to remove sequences containing ambiguous bases, barcode mismatches, or low quality reads (Phred quality scores < 25), a total 636,876 16S rRNA reads were obtained, with an average of 28,949 sequences per sample. After removing the singletons, there were still 25,328 sequences per sample. The minimum number of sequences reached for any of the samples simultaneously was 16,000; therefore, this value was used as the number of sequences to be generated in the rarefaction process. In this way, a standardized BIOM file was obtained for all samples. The depth of coverage in terms of the number of bacterial taxa per participant was adequate, since the tendency to asymptote was achieved in each curve. No significant differences were found in the rarefaction curves of phylogenetic diversity (*p* = 0.88) nor in the principal coordinate analysis (PCoA) of fecal communities (*p* = 0.139) when unweighted Unifrac distances were estimated, between the infected and uninfected groups.

**Fig. 1:**
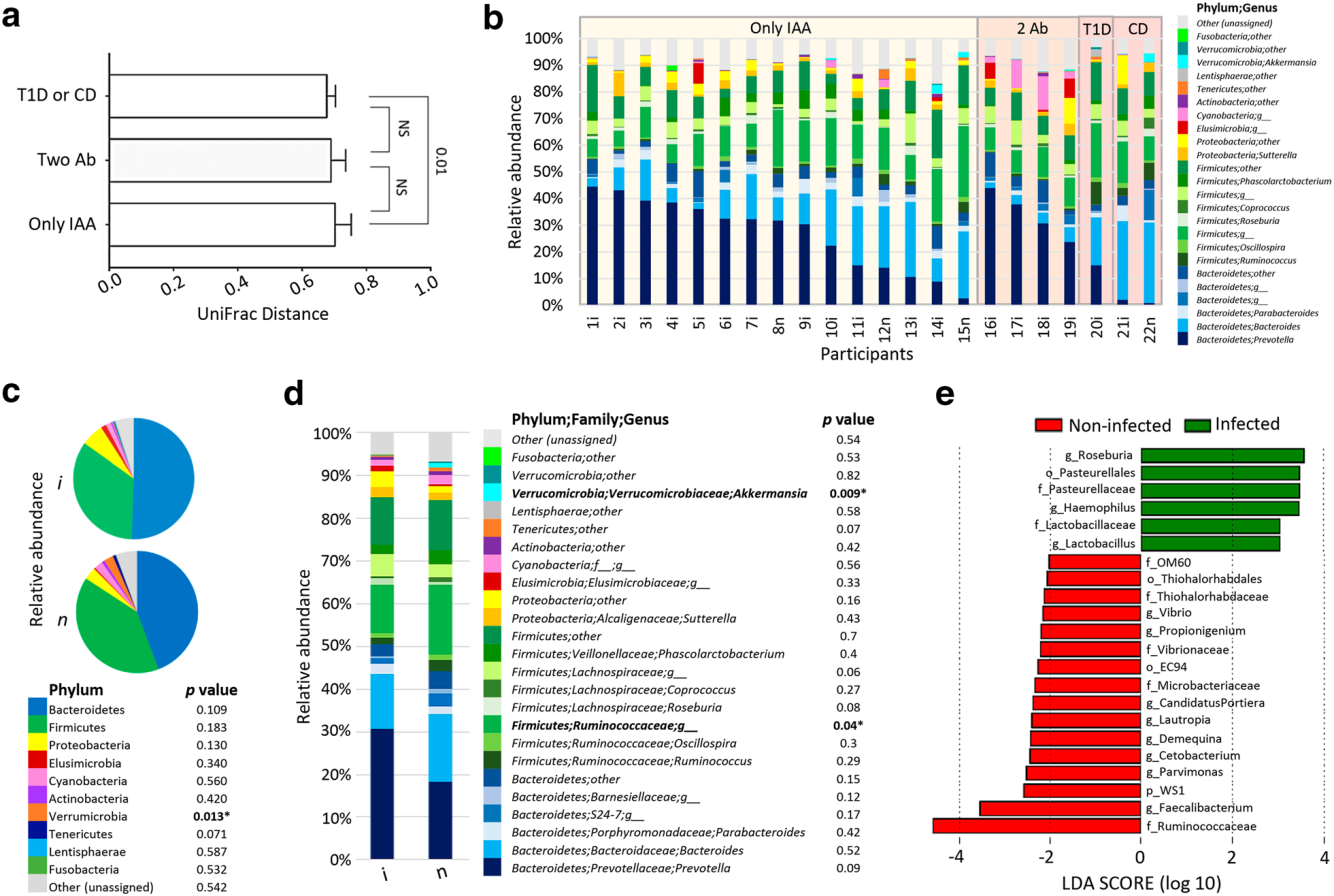
Fecal microbiota structure in Mexican children with positive T1D- or CD-associated antibodies (n = 22). **a** Weighted UniFrac distances between groups. **b** Bacterial relative abundance at genus level per participant. **c** Bacterial relative abundance according to their infection status by enteropathogenic parasites at phylum level and **d** at genus level (all phyla and genus with ≥ 1% of relative abundance were considered). **e** Linear Discriminant Analysis Effect Size (LEfSe) analysis is showing those OTUs that were significantly differentially abundant between autoimmune children infected or non-infected with *Cyptosporidium* and/or *Cyclospora*, ranked by effect size (all LDA scores > 2). Only IAA: Positives only for T1D-associated anti-insulin autoantibodies, Two Ab: Positives for 2 T1D and CD-associated autoantibodies, T1D: Type 1 diabetes, CD: Celiac disease, n: non-infected, i: infected with *Cyptosporidium* and/or *Cyclospora*, NS: non-significant (Student’s t test), *p* values ≤ 0.05 were considered significant

Otherwise, when plotting the PCoA of fecal bacterial communities in participants with diagnosis of T1D or CD (n = 3) against those with only one auto-antibody type (n = 15), the already diagnosed participants had a lower bacterial diversity within group (*p* = 0.01) than between groups. In the same analysis, autoimmune participants (with more than two positive autoantibodies for T1D), had a similar distribution of their UniFrac distances (*p* > 0.05) to those who were diagnosed with T1D or CD, as shown in Fig. 1a. However, no differences were found in alpha diversity (*p *> 0.05), evaluated as rarefaction curves of phylogenetic diversity among the three groups.

Figure 1b shows the relative bacterial genus abundance in the fecal microbiota of the 22 evaluated participants. *Prevotella* was the dominant enterotype (relative abundance of 30–45%) in samples from 12 participants; 9 with only AAI autoantibodies, 1 with autoimmunity but not symptomatic yet and 2 with anti-TG. Seven samples presented more than 20% of *Bacteroides* abundance, 2 of them from participants at CD onset where one was infected with three enteropathogenic parasites, and the other was uninfected. The rest of the sequenced samples corresponded to 5 participants with only IAA antibodies. Independently of enteropathogenic parasites, the group of participants with T1D or CD (samples 20i, 21i and 22n), presented an increased (*p *= 0.0001) relative abundance of Ruminococcus (5.99%) as compared to the abundance of the only IAA (1.41%) or 2 Ab (0.65%) groups. In addition, its Bacteroides relative abundance is also significantly higher (*p *= 0.001) than it of the 2 Ab group (Fig. 1b).

When assessing the individual effect of enteropathogenic parasites infection on bacterial diversity, no differences were detected in the rarefaction curves. However, participants with *Cryptosporidium* spp. tended (*p* = 0.057) to have lower bacterial diversity than the uninfected ones, while the *p* value was higher (*p* = 0.07) in those participants with *Cyclospora* spp., as well as for co-infected participants with both parasites (*p *= 0.064). Additionally, it highlights that parasitized participants with *Cryptosporidium* spp. and/or *Cyclospora* spp. had a lower (*p* > 0.0001) intragroup variability than non-infected children, being its most representative genera *Roseburia, Lactobacillus* and *Haemophilus*, as shown in Linear Discriminant Effect Size Analysis (LEfSe), schematized in Fig. 1e.

**Fig. 2:**
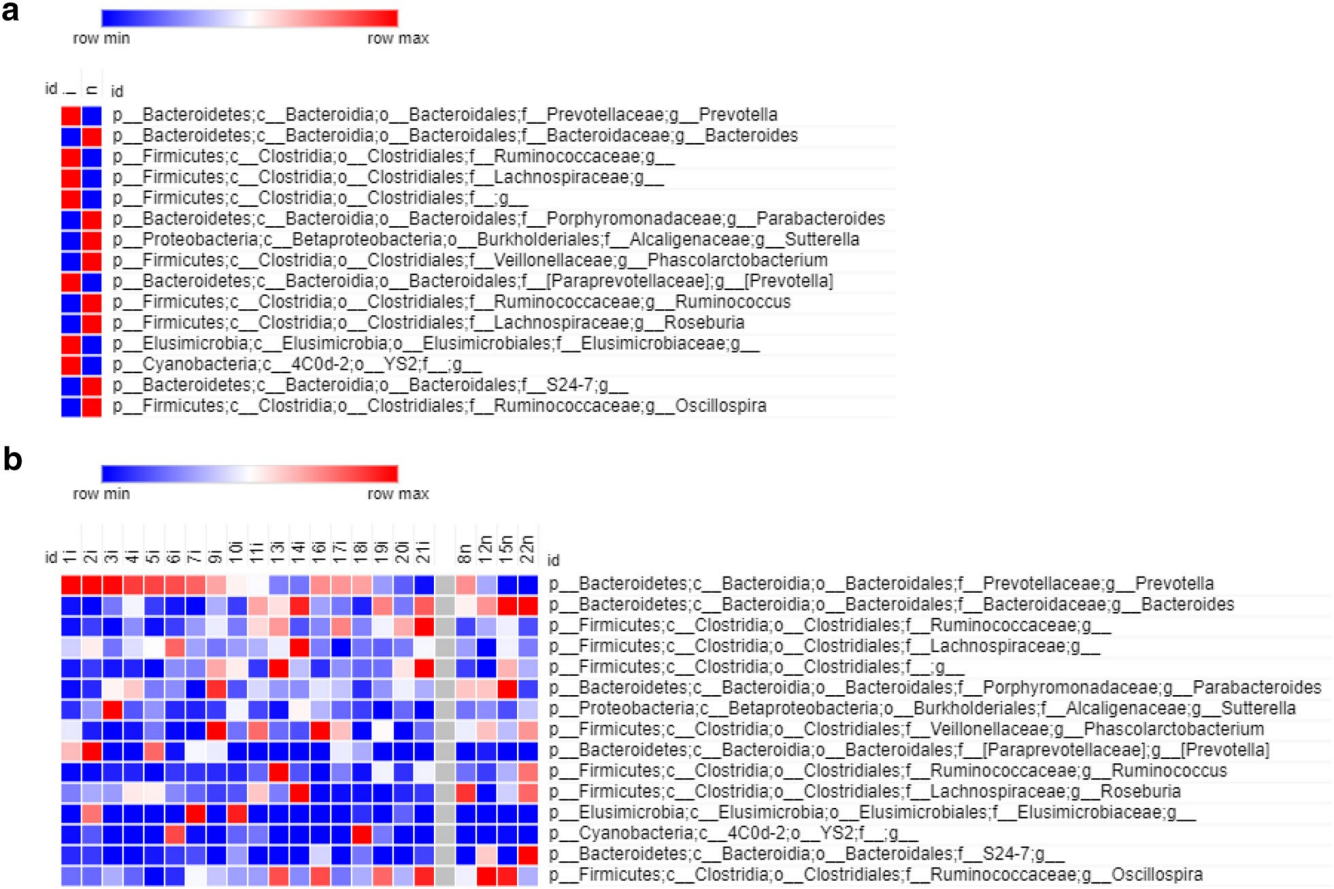
Heatmap analysis of gut microbiota. **a** Bacterial relative abundance means, in infected or non-infected children and **b** per participant. All OTUs with relative abundance greater than 0.01% were considered. *i* infected, *n* non infected

At the Phyla level, *Cryptosporidium* spp. and/or *Cyclospora* spp. infected participants had a lower abundance of Verrucomicrobia (*p* = 0.013) in feces than the uninfected ones (0.15 vs. 1.29%), as shown in Fig. 1c. This was explained by an increased abundance of the genus *Akkermansia* (*p* = 0.009) in uninfected participants when compared to the infected ones (1.21% ± 1.52 vs. 0.1% ± 0.21), as shown in Fig. 1d. In the same way, the Ruminococcaceae bacterial family from phylum Firmicutes, was more abundant (*p* = 0.04) in participants without *Cryptosporidium* spp. and/or *Cyclospora* spp. infections in comparison to the infected ones (16.36% ± 6.38 vs 11.36% ± 4.3). It was the operational taxonomic unit (OTU) with a higher linear discriminant analysis (LDA) score in the LEfSe analysis for that group (Fig. 1c, d). The Firmicutes/Bacteroidetes ratio was higher (*p *= 0.02) in uninfected participants in contrast with those with *Cryptosporidium* spp. and/or *Cyclospora* spp. (0.961 ± 0.142 vs. 0.653 ± 0.048). Figure 2 shows the visualization of the most abundant bacterial genera on a heatmap.

## Discussion

Damage of the gut mucosa epithelial barrier by parasites or other factors that affect permeability has been associated with the onset of autoimmune diseases, such as CD and T1D. Also, changes in the gut bacterial composition related to short-chain fatty acid production are involved in the same problem [11, 23, 36]. Therefore, we hypothesize that enteropathogenic parasites adhered or internalized in epithelial cells in the lining of the small intestine of high-risk children, who are developing autoimmunity, can induce bacterial imbalance and trigger the disease onset. This fact could be significant in children populations with a high prevalence of enteropathogenic parasites, such as the investigated Mexican population.

Firstly, our participants with high risk or already suffering from T1D or CD were highly parasitized. In our sampled children, infections with *Cryptosporidium* spp were present more than twice than the ones previously found in children from an open population in the same area [27]. Although cryptosporidiosis is common in our area, *C. parvum* and *C. hominis* infect children principally under five years old [34], and most of our participants were older than ten years. *Blastocystis* spp and *C. cayetanenesis* were also higher, and *G. lamblia* was similar to the reported data. The increased parasitism may be due to the detection method used (qPCR), which is more sensible than the conventional microscopy used in previous studies. However, parasites can opportunistically infect children with altered immunity [6, 38].

Thus, in a subsample of participants, the first part of our hypothesis was contrasted, although it is not possible to separate the effect of autoimmunity itself from the effect of parasitism. We found that the fecal bacterial microbiota structure of studied participants was significantly affected by the autoimmunity status (p = 0.012), in agreement with our previous study [21], as well as, because of the infection by *Cryptosporidium* spp. alone (p = 0.027) or in co-infection with *Cyclospora* spp. (p = 0.035). There were no confusion factors, as first year of life and current diet, genetic predisposition, or antibiotic use, involved for such effects to explain the microbiota differences between our groups (*p *> 0.05).

The effect of infection by *Cryptosporidium* spp. and *Cyclospora* spp. on the abundance and structure of some intestinal bacteria is poorly studied. Their previous life forms before going inside of the epithelial cells may interact with the intestinal lumen components, affecting the bacterial microbiota composition [28]. Alternatively, β-defensins 1 and 2, produced by the immune system against the parasites’ infection, may also induce changes in the bacterial composition [16].

The prevalence of *Blastocystis* spp. infection in our population appears high, although it is unclear if it is a pathogen, a commensal or an opportunistic parasite [6], because it can induce intestinal illness, but it can also produce an asymptomatic or self-limited course in the host. Possibly that was the reason why Audebert et al. [5] found effects of *Blastocystis* spp. infection on α and β bacterial diversity in human beings, whereas Nagel et al. [22] associated the bacterial changes to the irritable bowel syndrome of their patients. In agreement with Andersen et al. [4], we found that *Blastocystis* spp. in some fecal samples of infected participants were concomitant with a high relative abundance of *Ruminococcus* and *Prevotella*.

*Giardia lamblia* adheres to the epithelial cells in the small intestine, damaging the intestinal mucosa in a similar spectrum to the one produced by CD [26, 32]. Therefore, giardiasis induces transitory intolerance to gluten proteins, which can evolve to CD in genetically predisposed individuals [10, 24]. There is evidence of the onset of CD after giardiasis [10], and eventually, this infection could increase even more the risk of CD in children with T1D.

According to Maffeis et al. [20], both, the altered intestinal permeability and dysbiosis, are contemporaneously associated with the pre-pathogenic period of T1D condition. From our results, we could infer that infections of intracellular *Cryptosporidium* spp. and/or *Cyclospora* spp. could induce both increased gut permeability and dysbiosis, at least in our participants. We knew from our previous study [21] that dysbiosis was very strong in children at onset of T1D, with an increase in *Bacteroides* abundance. However, we had no information of the previous state (presence of autoantibodies or autoimmunity) in relation to dysbiosis, or about the effect of protozoan parasitic infection of our participants.

The microbiota structure of participants with just AAI antibodies, was similar to that of healthy participants, as well as to the ones of more than 2 years with T1D in our previous study, respecting *Prevotella* and *Bacteroides* proportions [21]. An interesting detail is that asymptomatic participants with two antibody types, presented about 6% Elusimicrobia and > 10% Cianobacteria, genera that was not found before in previous analyzed samples from the same population. Besides an increased abundance of Bacteroides, we also found an increased abundance of Ruminococcus (5.99%) in symptomatic participants with T1D or CD. Although data of bacterial species were not accessible in this study,, it could be due to *Bacteroides dorei* and *Ruminococcus gravus* found out by Abdellatif et al. [1], prior to the autoimmunity development.

It is remarkable that participants with no *Cryptosporidium* spp. and/or *Cyclospora* spp. infections had higher relative abundance of *Akkermansia* than infected participants. Hu et al. [16] demonstrated that *Akkermansia* promotes mucin production to protect the intestinal epithelium of the enteropathogenic parasites’ infection, and increases an antimicrobial peptide, Reg3γ [14]. *Akkermansia* is related also to the delay of T1D onset in a murine model [15]. Possibly, our uninfected children were protected by *Akkermansia* because they were mainly participants with only AAI positive.

The higher Firmicutes/Bacteroidetes ratio of the uninfected participants from our study, when compared to those infected with *Cryptosporidium* spp. and/or *Cyclospora* spp. could be explained, in part, by a higher amount of the Firmicutes’ Ruminococcaceae family in the uninfected participants. However, at least in a murine model, cryptosporidiosis was associated with an increased relative abundance of Bacteroidetes such as the Prevotellaceae and Porhyromondaceae families [28].

Only three of the infected participants with *Cryptosporidium* spp. and/or *Cyclospora* spp., referred gastrointestinal symptoms even though two of them were positive for CD-associated autoantibodies. Perhaps it was due to the fact that Lactobacillaceae family and *Lactobacillus* genera, distinctive of the infected participants (Fig. 1e), protected them from a severe affection. According to Sanad et al. [29], in a murine model, probiotics such as *Lactobacillus* and *Bifidobacterium* decreased the number of oocysts of *Cryptosporidium* spp. in feces, resulting in a reduced damage. However, induced cryptosporidiosis in other murine model [28] did not affect the same Phyla as in our *Cryptosporidium*-infected children, as expected due to the physiological differences.

## Conclusion

In conclusion, the prevalence of enteropathogenic parasites in northwestern Mexican children developing autoimmunity associated to T1D or CD is high; and particularly, *Cryptosporidium* spp. and/or *Cyclospora* spp. infection affected their fecal bacterial structure. The Firmicutes/Bacteroidetes ratio was modified by enteropathogenic parasites’ infection since the Verrucomicrobiaceae and Ruminococcaceae families were increased in uninfected children.

## Methods

### Characteristics of the Participants

The convenience non-probabilistic sampling study was carried out collecting data from 2016 to 2018 in northwestern Mexico (Sonora State), a desertic area with dry climate, previously described [2]. Children under 18 years old with a diagnosis of T1D or CD were recruited at the external consultation of the Children Sonora State Hospital and the Mexican Social Security Institute (HIES and IMSS). Additionally, children (7–12 years old) with autoantibodies related to T1D or CD were enrolled from public schools in the same geographical areas. Autoantibodies detection was done after an invitation to the schools’ principals, teachers and parents was made. A screening protocol was applied beginning with genotyping of HLA-DQ2 and DQ8 haplotypes, followed by familiar clinical history of risk, and ending with the autoantibodies’ detection.

After the parents’ consent and confirmation of no antibiotic or anti-parasites use in the last month, all participants were asked for one blood sample and feces samples of 3 consecutive days. The study was approved by the Ethical Committee of the Centro de Investigacion en Alimentacion y Desarrollo, A.C. (CE/016/2014) and an informed consent was signed by the parents. Positive and negative infected groups of participants with enteropathogenic parasites were formed for analysis.

Dried blood spots were used for gDNA extraction and HLA-DQ2 and DQ8 haplotypes analyses according to our technique [3]. After blood serum separation, antibodies analyses were performed. T1D associated autoantibodies, such as anti-insulin (AAI), anti-glutamate decarboxylase (GAD) and anti-tyrosin pancreatic phosphatase (IA-2) were analyzed by ELISA through commercial kits (MyBiosource, Cat. No. MBS772002 and Kronus, Cat. No. KR7770) or related to CD, such as anti-transglutaminase (TG) and anti-gliadins, by the ELISA method of Cabrera-Chávez et al. [7].

### Clinical history, diet, anthropometry, and socioeconomic status

Clinical history and socioeconomic information were obtained from hospital data and mother interviews. Three 24-h non consecutive recalls collected dietary intake data. Weight and height were measured.

### Enteropathogenic parasites identification

The three feces samples were homogenized together and aliquots were stored at − 70 °C for posterior analyses. Genomic DNA (gDNA) was extracted from fresh feces samples using the QIAmp Fast DNA stool mini kit (Qiagen, Cat. No. 51604), according to the manufacturer’s instructions. DNA was eluted in a final volume of 200 μL and stored at 20 °C. Concentration and quality were measured in a Nanodrop 2000 (Thermo Scientific, Pittsburgh, USA).

Feces’ gDNA samples were analyzed for enteropathogenic parasites *Cryptosporidium* spp., *Blastocystis* spp., *Cyclospora* spp., *Entamoeba histolytica (E. histolytica)* and *Giardia lamblia* (*G. lamblia*) by real-time PCR using specific primers for fragments in the 16S-like rRNA gene. Reactions were done with 5 μM of each primer, 10 uL of SYBR Green Supermix (Bio-Rad, Cat. No. 1708882), 100 ng of template DNA, and milli-Q water up to 20 μL final volume. Reactions were run in a StepOnePlus real-time PCR system (Applied Biosystems, Foster City, USA), to obtain the melting temperature (Tm) of each amplicon. Cycling conditions were different for each pathogen. For *Cyclospora* spp., conditions began with an initial hold at 95 °C for 10 min, followed by 40 cycles at 95 °C for 15 s and 60 °C for 1 min [37]. For *Cryptosporidium* spp., conditions were an initial 95 °C for 3 min, followed by 50 cycles at 94 °C for 15 s, 54 °C for 30 s and 72 °C for 30 s [18]. The *Blastocystis* spp. identification was done according to Grabensteiner and Hess [12], starting with an initial hold at 95 °C for 15 min, followed by 40 cycles at 94 °C for 30 s, 60 °C for 30 s and 72 °C for 60 s. For the *E. histolytica,* the conditions were the same previously described by ourselves [2]. For *G. lamblia*, the conditions were an initial 94 °C for 5 min, followed by 35 cycles at 94 °C for 30 s, 58 °C for 90 s, 72 °C for 90 s and 72 °C for 10 min [40].

### Bacterial microbiota analysis of feces

The extracted fecal gDNA of a subsample of 22 participants was used for the bacterial microbiota analysis. For this evaluation, the V3-V4 region of the bacterial 16S rRNA was amplified by PCR using the primer pair (S-D-Bact-0341-b-S-17/S-D-Bact-0785-a-A-21) [17], using the Illumina Miseq system (San Diego, CA., USA).

### Bioinformatics and statistical comparisons

Descriptive statistics were used for the characteristics of the participants. Student’s t test evaluated the diet composition between the infected and uninfected groups. Analyses were run in NCSS Statistical Software version 2007 considering significant *p* values ≤ 0.05.

The sequence analysis of the diversity of the microbial communities was made with the QIIME software package (Quantitative Insights Into Microbial Ecology), version 1.8.0 [8]. Quality filters were used to remove sequences containing ambiguous bases, barcode mismatches, or low quality reads (Phred quality scores < 25). After trimming barcodes, the sequences were clustered using UCLUST into operational taxonomic units (OTUs), based on 97% sequence similarity against the Green Genes reference sequence collection (version 13_5). The option of reverse strand matching was enabled. We selected the closed-reference OTU picking method that was a reference-based approach. Thus, chimera removal was not necessary. All analyses were restricted to OTUs comprising ≥ 0.1% of the reads. Weighted UniFrac distances and Principal Coordinate Analysis were used for the analysis of bacterial communities and comparisons [19]. Relative abundances for phylum, class, order and family were obtained and plotted in Excel. Genus whose relative abundance was greater than 1% were represented on a heat map; the visualization was carried out with the Morpheus software (https://software.broadinstitute.org/morpheus/). A linear discriminant analysis was performed to measure the effect (LEfSe) of the statistical significance of the obtained results with the biological consistency and to estimate the size of the effect [31]. Differences between groups were evaluated by ANOVA and Tukey–Kramer multiple comparison tests.

## Data Availability

The datasets analyzed during the current study are available from the corresponding author on reasonable request.
